# Doxycycline reduces the migration of tuberous sclerosis complex-2 null cells - effects on RhoA-GTPase and focal adhesion kinase

**DOI:** 10.1111/jcmm.12593

**Published:** 2015-08-18

**Authors:** Ho Yin Ng, Brian Gregory George Oliver, Janette Kay Burgess, Vera P Krymskaya, Judith Lee Black, Lyn M Moir

**Affiliations:** a Sydney Medical School, Discipline of Pharmacology, University of SydneySydney, NSW, Australia; b Cell Biology Group, Woolcock Institute of Medical ResearchSydney, NSW, Australia; c Department of Medicine, University of PennsylvaniaPhiladelphia, PA, USA; d Abramson Cancer Center, University of PennsylvaniaPhiladelphia, PA, USA; e School of Medical and Molecular Biosciences, University of Technology SydneySydney, NSW, Australia

**Keywords:** pulmonary lymphangioleiomyomatosis, tuberous sclerosis gene complex-2, doxycycline, rapamycin, migration, wound closure, RhoA-GTPase, focal adhesion kinase

## Abstract

Lymphangioleiomyomatosis (LAM) is associated with dysfunction of the tuberous sclerosis complex (TSC) leading to enhanced cell proliferation and migration. This study aims to examine whether doxycycline, a tetracycline antibiotic, can inhibit the enhanced migration of TSC2-deficient cells, identify signalling pathways through which doxycycline works and to assess the effectiveness of combining doxycycline with rapamycin (mammalian target of rapamycin complex 1 inhibitor) in controlling cell migration, proliferation and wound closure. *TSC2*-positive and *TSC2*-negative mouse embryonic fibroblasts (MEF), 323-*TSC2*-positive and 323-*TSC2*-null MEF and Eker rat uterine leiomyoma (ELT3) cells were treated with doxycycline or rapamycin alone, or in combination. Migration, wound closure and proliferation were assessed using a transwell migration assay, time-lapse microscopy and manual cell counts respectively. RhoA-GTPase activity, phosphorylation of p70S6 kinase (p70S6K) and focal adhesion kinase (FAK) in *TSC2*-negative MEF treated with doxycycline were examined using ELISA and immunoblotting techniques. The enhanced migration of *TSC2*-null cells was reduced by doxycycline at concentrations as low as 20 pM, while the rate of wound closure was reduced at 2–59 μM. Doxycycline decreased RhoA-GTPase activity and phosphorylation of FAK in these cells but had no effect on the phosphorylation of p70S6K, ERK1/2 or AKT. Combining doxycycline with rapamycin significantly reduced the rate of wound closure at lower concentrations than achieved with either drug alone. This study shows that doxycycline inhibits *TSC2*-null cell migration. Thus doxycycline has potential as an anti-migratory agent in the treatment of diseases with TSC2 dysfunction.

## Introduction

Pulmonary lymphangioleiomyomatosis (LAM), a rare disease in women of childbearing age 1,2, is characterised by the irregular proliferation and migration of smooth muscle-like cells (LAM-cells) throughout the lungs, resulting in the obstruction of the small airways 3,4. The disease ultimately manifests as cystic destruction of the lung parenchyma and the loss of pulmonary function 5.

Lymphangioleiomyomatosis is associated with the mutational inactivation of the tuberous sclerosis gene complex (TSC), *TSC1* and *TSC2* 6,7. Dysfunction of either TSC1 (hamartin) or TSC2 (tuberin; with TSC2 being more common) results in enhanced cell proliferation and migration 8–10. Over the last decade, knowledge of the underlying cellular mechanisms that drive LAM pathophysiology has been enhanced with an increased understanding of the rapamycin-sensitive, mammalian target of rapamycin complex 1 (mTORC1) 11 and the rapamycin-insensitive mTORC2 signalling pathways 12–15. The TSC1/TSC2 complex indirectly regulates the phosphorylation of ribosomal p70S6 kinase (p70S6K) and the initiation factor 4E-binding protein 1 (4E-BP1) through the mTORC1 pathway, thus acting as a central regulator of cell growth and proliferation 16–18. The functional TSC1/TSC2 complex also acts to regulate the mTORC2 pathway, which controls actin cytoskeleton rearrangement through Rho GTPases, RhoA and Rac1 19,20. Dysfunction of the TSC1/TSC2 complex leads to the activation of mTORC2, resulting in increased RhoA-GTPase activity and consequently enhanced cellular migration 21.

Cell migration in LAM has been demonstrated clinically, where it is reported that the same TSC2 mutation is observed in pulmonary LAM cells and angiomyolipoma (AML) cells 22. This suggests that the cells have originated from a common origin, disseminating through the vascular and lymphatic systems 23. This also may explain the recurrence of LAM cells in the healthy lungs received by LAM patients after lung transplant 24–28. *In vitro* studies by Goncharova *et al*. have also provided supporting evidence that a functional TSC1/TSC2 complex acts to regulate normal cell migration through the mTORC2 pathway, which controls RhoA-GTPase activity 21.

The mTORC1 inhibitor rapamycin, a bacterial macrolide with immunosuppressive and antitumour properties, has been a major focus of LAM research. *In vitro* studies have shown rapamycin to effectively inhibit LAM cell proliferation 9,29,30. In addition, in the Multicenter International LAM Efficacy of Sirolimus trial, McCormack *et al*. reported that patients with LAM treated with sirolimus (rapamycin) experienced stabilised lung function with a reduction in symptoms and improvement in quality of life compared to patients who were taking a placebo 31. However limitations were present, most notably the continued decline in lung function following the discontinuation of sirolimus 31. Similarly, in a murine model of TSC2-null tumours, rapamycin inhibited tumour growth but its withdrawal resulted in TSC2-null tumour regrowth together with a decreased survival 21. Although the long-term effects of rapamycin are not known in LAM, chronic sirolimus use has previously been associated with altered lipid and glucose metabolism resulting in hyperlipidaemia, glucose intolerance, diabetes like syndromes and cancer 32–35. Together, these *in vitro* and *in vivo* studies highlight that although rapamycin shows beneficial effects in the treatment of LAM, its requirement for long-term use and its potential adverse effects highlight the need for alternative treatments.

Since enhanced migration is a prominent feature of TSC2-null cells, the lack of effect of rapamycin on migration 9 suggests that mTORC1 inhibitors alone may not be the optimal treatment for LAM. Alternative treatments, in particular, drugs that tackle the increased migratory capacity of LAM cells may provide added benefit. Since LAM is a disease characterised by enhanced cell proliferation and migration, where both processes occur, the examination of the effects of drugs on both processes is necessary.

Moses *et al*. demonstrated that doxycycline, a second-generation tetracycline antibiotic, improved lung function and quality of life in a single LAM patient, with minimal side-effects 36. In addition, we have previously reported that doxycycline can reduce the mitochondrial activity and extracellular levels of active matrix metalloproteinase 2 (MMP-2) in human LAM cells and *TSC2*-null mouse embryonic fibroblasts (MEF) 37. Furthermore, doxycycline has been shown to inhibit migration of cells in the development of arterial intimal lesions 38, breast carcinoma 39 and human melanoma 40.

Since the loss of TSC2 plays a prominent role in the phenotypic characteristics in LAM, the study of cells that are well characterised for TSC2 dysfunction is highly relevant. Examples of these cells are the TSC2 knockout MEF 41, 323 MEF in which TSC2 is stably reintroduced 13 and the Eker rat uterine leiomyoma (ELT3) cells in which TSC2 is naturally absent 42.

In this study, we investigated the effects of doxycycline on the migratory capacity of cells deficient for TSC2, identified the signalling pathways through which doxycycline works and examined the potential of combining doxycycline with the mTORC1 inhibitor rapamycin, to inhibit the enhanced migration, proliferation and wound closure of *TSC2*-deficient cells.

## Materials and methods

### Cell culture

Littermate derived p53 knockout *TSC2*-negative MEF, *TSC2*-positive MEF and isogenic p53 positive 323-*TSC2*-null MEF and 323-*TSC2*-positive MEF (where TSC2 was stably reintroduced) (a gift from Dr. D. Kwiatkowski, Brigham and Women's Hospital Boston, MA, USA) and ELT3 cells that were spontaneously deficient in TSC2 (a gift from Dr. Cheryl Walker, MD Anderson Cancer Center, TX, USA) were cultured as previously described 10,11,21,42,43.

### MEF, 323 MEF and ELT3 cell preparation

For experiments with MEF and 323 MEF, the cells were seeded at a density of 1 × 10^4^ cells/cm^2^ in high glucose DMEM (Life Technologies, Carlsbad, CA, USA) containing 10% foetal bovine serum (FBS - Glendarach Biological, Melbourne, VIC, Australia) and 1% penicillin-streptomycin (Life Technologies) for 24 hrs. The medium was then changed to DMEM containing 0.5% FBS and 1% penicillin-streptomycin (serum-reduced DMEM) for 24 hrs. The cells were then used as indicated in the experiments.

ELT3 cells were cultured in DF8 medium (supplemented with 10% FBS) as previously described 10,11,42,43. ELT3 cells were seeded at a density of 1 × 10^4^ cells/cm^2^ in DF8 medium for 24 hrs, after which the medium was changed to serum free DF8-basal medium for 24 hrs as previously described 11. The cells were then used as indicated in the experiments.

### Doxycycline, rapamycin and Y-27632 preparation

Doxycycline hyclate (Sigma-Aldrich, St. Louis, MO, USA) was freshly prepared in sterile distilled water at a stock concentration of 20 mM. Insolution^™^ rapamycin (Calbiochem, St. Louis, MO, USA) was diluted in sterile dimethyl sulphoxide (DMSO; Sigma-Aldrich) to a stock concentration of 1 mM and stored at −20°C prior to use. Y-27632 (Calbiochem) a ROCK inhibitor was diluted in sterile water to a stock concentration of 10 mM and stored at −20°C prior to use.

For all experiments, doxycycline was used at a final concentration range 200 fM–59 μM, rapamycin at 0.2–200 nM (with respective DMSO vehicle controls) and Y-27632 at 0.3–30 μM. For experiments in which the effects of combining doxycycline and rapamycin were assessed, various combinations of concentrations were used as indicated, with respective DMSO vehicle controls.

### Proliferation assay

Cells that were seeded and placed under serum-reduced conditions in 12-well flat-bottom, tissue-culture treated polystyrene cell culture plates (BD Falcon^™^; Becton Dickinson, Franklin Lakes, NJ, USA) as described above, were pretreated with doxycycline or rapamycin alone or in combination or with Y-27632 in the presence of serum-reduced DMEM for 30 min. The cells were subsequently stimulated with 10% FBS with or without drug for 24 hrs. Cells were then trypsinsed and proliferation was assessed by counting the number of viable cells (trypan blue exclusion) using a haemocytometer.

### Migration assay

Cells that were seeded and placed in serum reduced conditions in 75 cm^2^ tissue culture flasks (BD Falcon^™^; Becton Dickinson) as described above were trypsinised, counted and resuspended in serum-reduced DMEM. Cell migration was assessed using a transwell migration assay as previously described 8,10. Briefly, cells in suspension were pre-treated with doxycycline or rapamycin alone or in combination, or with Y-27632 (ROCK inhibitor) for 30 min before seeding onto collagen type I (1 μg/ml) coated cell culture inserts (8.0 μm polyethylene terephthalate membrane; BD Falcon^™^; Becton Dickinson) at a density of 2 × 10^5^ cells per 0.3 cm^2^. The cells were then allowed to migrate for 4 hrs towards a chemoattractant of 10% FBS at 37°C, 5% CO_2_. Non-migrated cells were removed and migrated cells were fixed with 4% (v/v) paraformaldehyde, stained with 0.5% (w/v) toludine blue (Sigma-Aldrich) containing 0.5% (w/v) boric acid (Sigma-Aldrich) and imaged (Olympus BX60; Olympus, Center Valley, PA, USA). Cell migration was measured by manually counting cells in five regions of 200 μm^2^ and expressed as an average.

### Wound assay

Cells that were seeded and placed under serum-reduced conditions in 12-well flat-bottom polystyrene cell culture plates precoated with collagen type I (1 μg/ml) as described above, were pre-treated with doxycycline or rapamycin alone, or in combination for 30 min prior to wounding. A wound was created by scratching the cell layer with a sterile 200 μl pipette tip (Thermo Fisher Scientific, Waltham, MA, USA). The cells were rinsed with serum-reduced DMEM to remove any cell debris and freshly prepared drug treatments were replaced into each well. The cells were then incubated at 37°C, 5% CO_2_ in a humidified incubation chamber (Clear State Solutions, Melbourne, VIC, Australia) mounted on a Nikon Eclipse Ti-microscope (Nikon Eclipse Ti, Tokyo, Japan). Time-lapse images were captured at intervals of 1 hr for up to 20 hrs as indicated and the rate of wound closure was assessed using Nikon Imaging software (NIS-Elements Imaging Software Version 3.22.01, Melville, NY, USA).

### Immunoblotting

Levels of phosphorylated (phospho)-p70S6 kinase (p70S6K), phospho-AKT, phospho p44/42 MAPK (ERK1/2) and phospho-focal adhesion kinase (FAK) were measured using immunoblotting as previously described 44. Briefly, cells that were seeded and placed in serum-reduced conditions in 6-well flat-bottom polystyrene cell culture plates were treated with doxycycline or rapamycin alone or in combination or with Y-27632. The cells were then lysed in modified RIPA buffer and the samples were size fractionated on 10% polyacrylamide gels and transferred to polyvinvylidene fluoride membranes (Millipore, Billerica, MA, USA). The membranes were then blocked with 5% bovine serum albumin (BSA) diluted in PBS with 0.05% Tween-20 (PBS-T) and subsequently incubated with respective primary antibodies against phosphorylated proteins (as indicated in Table1) overnight at 4°C, before incubation with respective immunoglobulin G-horseradish peroxidase (HRP) conjugated secondary antibody (as indicated in Table1) for 1 hr at room temperature. The membranes were then visualised using chemiluminescence (Millipore, Temecula, CA, USA), imaged and analysed using a Kodak Image Station 4000MM (Carestream Molecular Imaging Software Version 5.0.2.30, Rochester, NY, USA).

**Table 1:** Antibodies for immunoblotting

Target protein	Primary antibody/Concentration (supplier)*	Secondary antibody (supplier)*	Incubation time (hrs)
p70S6K	Rabbit polyclonal anti-mouse Phospho-p70S6K (Thr^389^)/1:2000 (Cell Signalling Technology, Danvers, MA, USA)		1/2
	Rabbit polyclonal anti-mouse p70S6K/1:2000 (Cell Signalling Technology)		
AKT	Rabbit monoclonal anti-mouse Phospho-AKT (Thr^308^)/1:2000 (Cell Signalling Technology)		1/4
	Rabbit monoclonal anti-mouse AKT/1:2000 (Cell Signalling Technology)	Goat polyclonal anti-rabbit immunoglobulin G-HRP conjugated 1:2000 (Dako Glosturp, Denmark)	
p42/44 ERK1/2 MAPK	Rabbit monoclonal anti-mouse Phospho-p44/42 MAPK (ERK1/2) (Thr^202^/Thr^204^)/1:2000 (Cell Signalling Technology)		1/4
	Rabbit monoclonal anti-mouse p44/42 MAPK (ERK1/2)/1:2000 (Cell Signalling Technology)		
FAK	Rabbit polyclonal antimouse Phospho-FAK (Tyr^397^)/1:2000 (Cell Signalling Technology)		4
	Rabbit polyclonal antimouse FAK/1:2000 (Cell Signalling Technology)		
α-Tubulin	Mouse polyclonal α-tubulin/1:2000 (Santa Cruz Biotechnology)	Goat polyclonal antimouse immunoglobulin G-HRP conjugated 1:2000 (Dako)	–

* Primary and secondary antibodies were diluted in 1% BSA/PBS-T.

To allow for comparison of phosphorylated proteins to total proteins, antibodies were stripped from the membranes, the membranes were re-blocked and re-probed as previously described with either primary antibodies against total proteins (as indicated in Table1) or mouse anti-α-tubulin (loading control; 1:2000 dilution in 1% PBS-T; Santa Cruz Biotechnology, Santa Cruz, CA, USA) 44. Respective immunoglobulin G-HRP conjugated secondary antibodies (as indicated in Table1) were used.

### RhoA activation assay

RhoA activity was measured using the G-LISA RhoA Activation Assay Biochem Kit as per the manufacturer's instructions (Cytoskeleton, Denver, CO, USA). Briefly, cells that were seeded and placed in serum-reduced conditions in 175 cm^2^ tissue cell culture flasks were treated with or without Y-27632, rapamycin or doxycycline alone or in combination as indicated for 1 min. (as assessed by time-course experiments to exhibit maximum RhoA expression levels). The cells were then lysed and the activity of RhoA was measured using the G-LISA assay at an absorbance of 490 nm with the limits of detection at 0.05 ng. Following the manufacturer's instructions, levels of active RhoA were then normalised to total Rho (-A, -B, -C) levels as measured using immunoblotting (as detailed above). Mouse monoclonal anti-Rho (-A, -B, -C) primary antibody (diluted 1:2000; Millipore) and goat polyclonal antimouse immunoglobulin G-HRP conjugated secondary antibody (diluted 1:2000; Dako) were used.

### Statistical analysis

Results from *n* experiments were analysed using Student's unpaired *t*-test, area under the curve, repeated measures one-way anova or repeated measures two-way anova with a Bonferroni post-test where appropriate (Graphpad Prism Version 4; Graphpad Software Inc, La Jolla, CA, USA). A probability (*P*) value of less than or equal to 0.05 was considered statistically significant.

## Results

### Enhanced cell migration and increased rate of wound closure in TSC2-deficient cells

**Figure 1:**
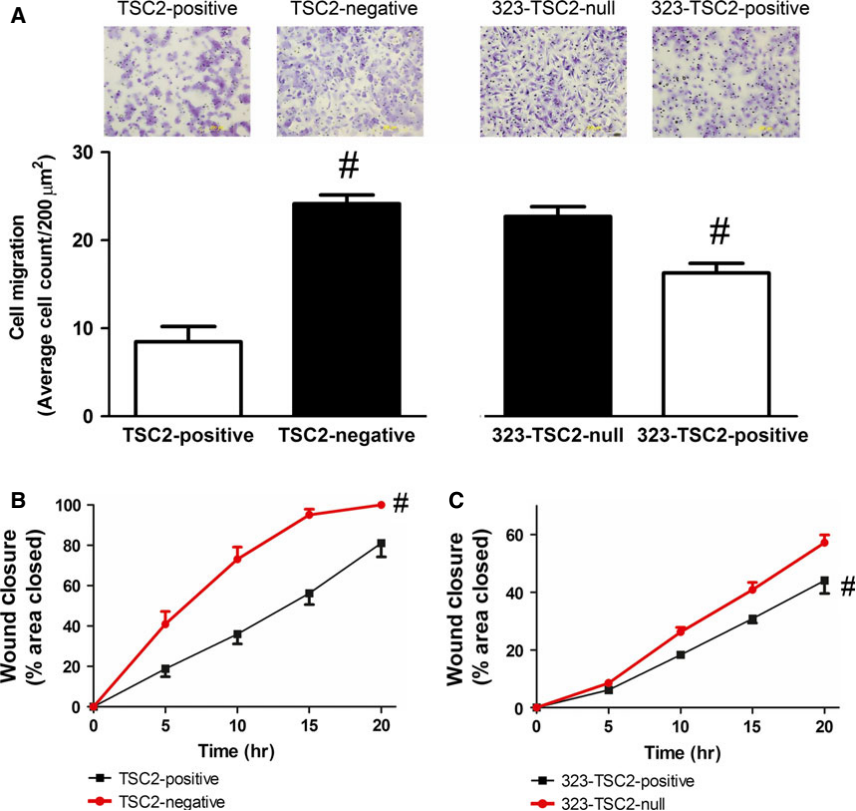
10% FBS-induced migration of (**A**) TSC2-positive (*n* = 10) and TSC2-negative (*n* = 10) MEF and 323-TSC2-positive MEF (*n* = 5) and 323-TSC2-null MEF (*n* = 5). Data represent the average number of cells migrated in five regions of 200 μm^2^ ± SEM, ^#^*P* ≤ 0.05 unpaired *t*-test. Wound closure assay of (**B**) TSC2-positive (black line, *n* = 4) and TSC2-negative MEF (red line, *n* = 4) over 20 hrs and (**C**) 323-TSC2-positive (black line, *n* = 4) and 323-TSC2-null MEF (red line, *n* = 4) over 20 hrs. Data expressed as a percentage closure ± SEM in response to 0.5% FBS stimulation, ^#^*P* ≤ 0.05 repeated measures one-way anova with a Bonferroni post test.

In this study, we confirmed the enhanced migration of p53 knockout TSC2-negative MEF compared to TSC2-positive MEF (TSC2-negative and TSC2-positive, *n* = 10; *P* ≤ 0.05, Fig.1A) 9,10 and extended our findings to show, using 323-TSC2-positive MEF in which TSC2 has been stably reintroduced, that cell migration was significantly lower in cells where TSC2 is present (323-TSC2-null and 323-TSC2-positive MEF, *n* = 5; *P* ≤ 0.05, Fig.1A). In addition, the rate of wound closure in TSC2-negative MEF was also significantly greater compared to TSC2-positive MEF (area under the curve, TSC2-negative and TSC2-positive, *n* = 4; *P* ≤ 0.05, Fig.1B). Again, using 323-TSC2-positive MEF, the rate at which the cells closed a wound was significantly decreased by the presence of TSC2 (323-TSC2-positive and 323-TSC2-null, *n* = 4; *P* ≤ 0.05, Fig.1C).

### Doxycycline reduced enhanced cell migration in TSC2-deficient cells

**Figure 2:**
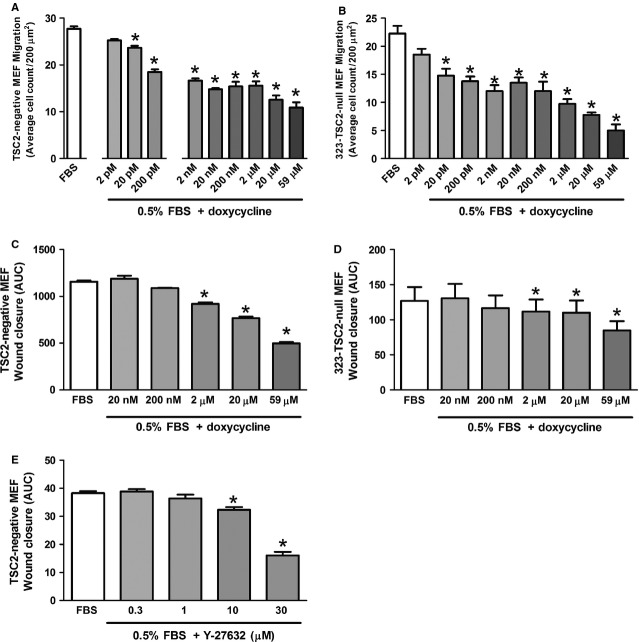
Migration of (**A**) TSC2-negative MEF (*n* = 5) and (**B**) 323-TSC2-null MEF following 30 min pre-treatment with doxycycline. Data expressed as the average number of cells migrated in five regions of 200 μm^2^ ± SEM. Wound closure assay of (**C**) TSC2-negative MEF (*n* = 4) and (**D**) 323-TSC2-null MEF (*n* = 4) following 30 min pre-treatment with doxycycline. Data expressed as area under the curve (AUC) over 20 hrs ± SEM. Migration of (**E**) TSC2-negative MEF (*n* = 4) following 30 min pre-treatment with Y-27632. Data expressed as the average number of cells migrated per 200 μm^2^, *P ≤ 0.05 repeated measures one-way anova compared with FBS with a Bonferroni post-test.

Doxycycline (20 pM–59 μM) decreased 10% FBS-induced migration of TSC2-negative MEF by 14.5–60.7% (TSC2-negative, *n* = 5; *P* ≤ 0.05, Fig.2A) and of 323-TSC2-null MEF by 33.7–77.5% (323-TSC2-null MEF, *n* = 5; *P* ≤ 0.05, Fig.2B). Doxycycline also decreased the rate of wound closure in both TSC2-negative MEF and 323-TSC2-null MEF (2–59 μM area under the curve, TSC2-negative and 323-TSC2-null MEF, *n* = 4; *P* ≤ 0.05, Fig.2C and D). Similarly, the ROCK inhibitor, Y-27632 (10–30 μM) decreased FBS-induced migration of TSC2-negative MEF by 15.6–58.0% (TSC2-negative MEF, *n* = 4; *P* ≤ 0.05, Fig.2E). In contrast, rapamycin, had no effect on the migration of TSC2-negative MEF and 323-TSC2-null MEF at any of the concentrations tested (2–200 nM; data not shown) in accordance with previous studies 9.

### Doxycycline and Y-27632 had no effect on cell proliferation

0.5% FBS and 10% FBS induced the proliferation of TSC2-negative MEF to a greater extent than TSC2-positive MEF (TSC2-negative and TSC2-positive, *n* = 12; *P* ≤ 0.05, Fig. S1) and this is in accordance with previous studies 11,45. Cell proliferation of 323-TSC2-positive MEF in the presence of 10% FBS was less than that in 323-TSC2-null MEF (323-TSC2-positive and 323-TSC2-null MEF, *n* = 8; *P* ≤ 0.05, Fig. S2).

**Figure 3:**
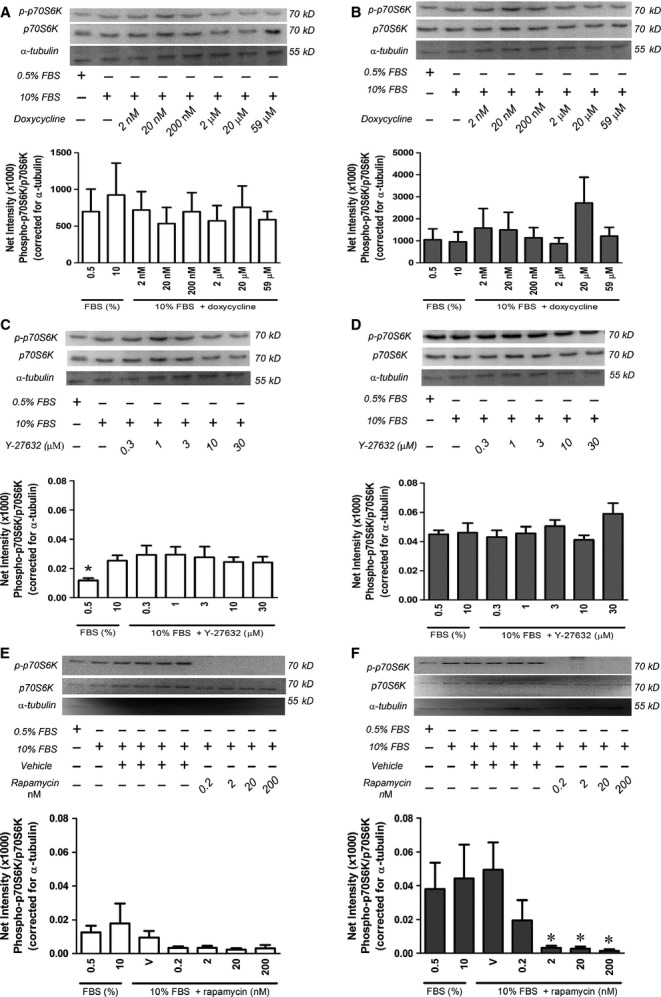
Phospho-p70S6K levels in (**A**) TSC2-positive MEF (*n* = 5) and (**B**) TSC2-negative MEF (*n* = 5) in the presence (+) or absence (−) of doxycycline as indicated (**C**) TSC2-positive MEF (*n* = 3) and (**D**) TSC2-negative MEF (*n* = 3) in the presence (+) or absence (−) of Y-27632 as indicated and (**E**) TSC2-positive MEF (*n* = 4) and (**F**) TSC2-negative MEF (*n* = 4) in the presence (+) or absence (−) of vehicle control (V) or rapamycin as indicated. Representative western blots of phospho-p70S6K, p70S6K and α-tubulin are shown (**A**–**F**) above mean data. Data expressed as mean ± SEM, *P ≤ 0.05 repeated measures one-way anova with a Bonferroni post-test (compared with 10% FBS).

We have previously shown that doxycycline has no effect on the proliferation of TSC2-positive or TSC2-negative MEF 37 and we were able to replicate these findings in 323-TSC2-positive and 323-TSC2-null MEF (323-TSC2-positive and 323-TSC2-null MEF, *n* = 5; *P* > 0.05, Fig. S3) and in ELT3 cells where doxycycline inhibited cell proliferation only at the highest concentration (59 μM; ELT3 cells, *n* = 5, *P* ≤ 0.05, Fig. S4). In addition, Y-27632 had no effect on the proliferation of TSC2-positive or TSC2-negative MEF (TSC2-positive and TSC2-negative MEF, *n* = 4; *P* > 0.05, Fig. S5). To confirm that proliferation in the above cell types can be inhibited, the mTORC1 inhibitor rapamycin was used as a positive control, showing a reduction in the proliferation of MEF, 323 MEF and ELT3 cells (TSC2-negative and TSC2-positive, *n* = 5; *P* ≤ 0.05, Fig. S6, 323-TSC2-positive and 323-TSC2-null MEF, *n* = 5; *P* ≤ 0.05, Fig. S7 and ELT3, *n* = 5; *P* ≤ 0.05, Fig. S8). Furthermore, doxycycline and Y-27632 had no effect on the phosphorylation of p70S6K (TSC2-positive or TSC2-negative, *n* = 5, *P* > 0.05, doxycycline Fig.3A and B, Y-27632 Fig.3C and D), whereas rapamycin inhibited the phosphorylation of p70S6K (TSC2-positive and TSC2-negative, *n* = 5, *P* ≤ 0.05, Fig.3E and F).

### Doxycycline inhibits RhoA activity and reduces phosphorylation of FAK in TSC2-negative MEF

**Figure 4:**
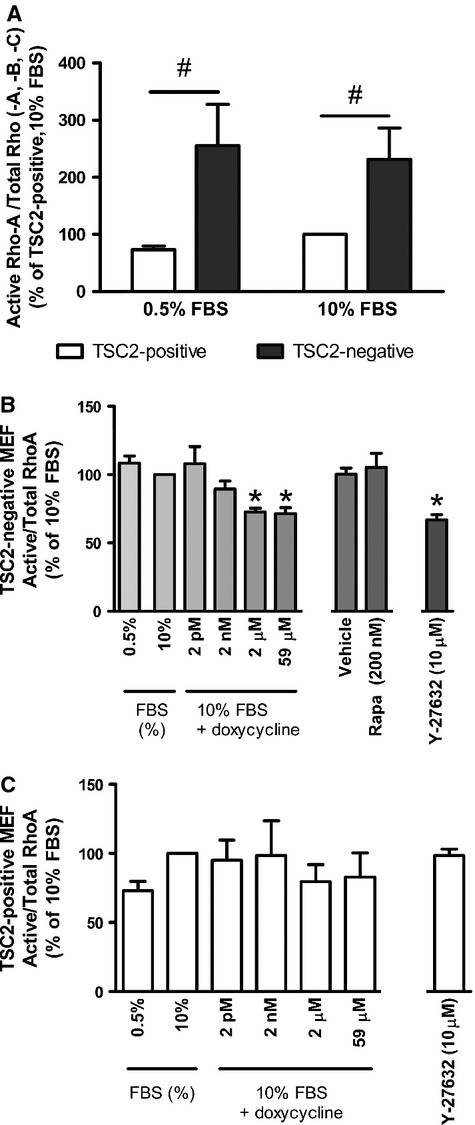
RhoA-GTPase activity in (**A**) TSC2-positive (*n* = 5) and TSC2-negative MEF (*n* = 6) under basal conditions (0.5% FBS) and in the presence of the stimulus 10% FBS, ^#^*P* ≤ 0.05 TSC2-positive *versus *TSC2-negative cells (**B**) TSC2-negative MEF (*n* = 6) following 1 min. of treatment with doxycycline, vehicle control (DMSO), rapamycin or Y-27632 as indicated and (**C**) TSC2-positive MEF (*n* = 5) following 1 min. of treatment with doxycycline, or Y-27632. RhoA activity corrected for total Rho (-A, -B, -C) and expressed as a percentage of 10% FBS ± SEM, *P ≤ 0.05 repeated measures one-way anova with a Bonferroni post-test (compared with 10% FBS).

To investigate the mechanism through which doxycycline inhibits the migration of TSC2-deficient cells, we examined the activity of RhoA-GTPase and the phosphorylation of FAK, AKT and ERK1/2. Under basal conditions and in the presence of 10% FBS, TSC2-negative MEF exhibited increased RhoA-GTPase activity compared to TSC2-positive MEF in accordance with published studies 43,46. In addition, in TSC2-positive MEF, RhoA-GTPase activity was induced in the presence of 10% FBS, whereas this was constitutively active in TSC2-negative MEF (TSC2-positive and TSC2-negative MEF, 0.5% FBS and 10% FBS, *P* ≤ 0.05, Fig.4A).

Doxycycline (2 and 59 μM) and Y-27632 (10 μM) reduced elevated RhoA-GTPase activity in TSC2-negative MEF by 26.8–34.3% and 38.4% at 1 min. (TSC2-negative, doxycycline and Y-27632, *n* = 6, *P* ≤ 0.05, Fig.4B), while rapamycin (200 nM) had no effect (TSC2-negative, rapamycin 200 nM, *n* = 6, *P* ≥ 0.05, Fig.4B). In addition, doxycycline had no effect on RhoA-GTPase activity in TSC2-positive MEF (TSC2-positive, *n* = 4, *P* > 0.05, Fig.4C).

**Figure 5:**
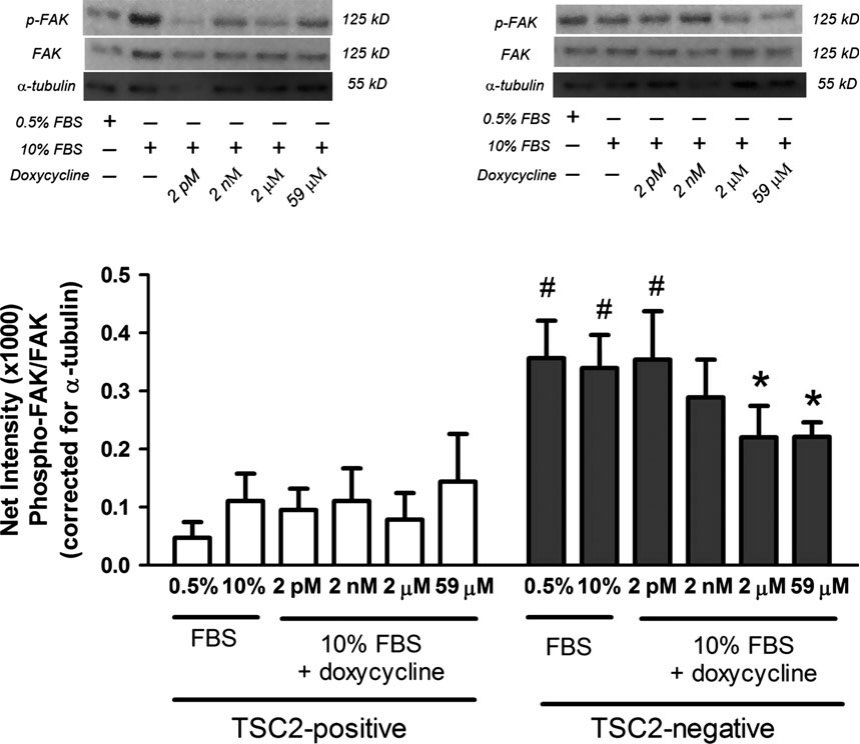
Phospho-FAK levels in TSC2-positive and TSC2-negative MEF (*n* = 4) in the presence (+) or absence (−) of doxycycline treatment for 4 hrs. Representative western blots of phospho-FAK, FAK and α-tubulin are shown above mean data. Data expressed as mean ± SEM, ^#^*P* ≤ 0.05 compared with TSC2-positive cells; *P ≤ 0.05 compared with 10% FBS in TSC2-negative cells using repeated measures one-way anova with a Bonferroni post-test.

**Figure 6:**
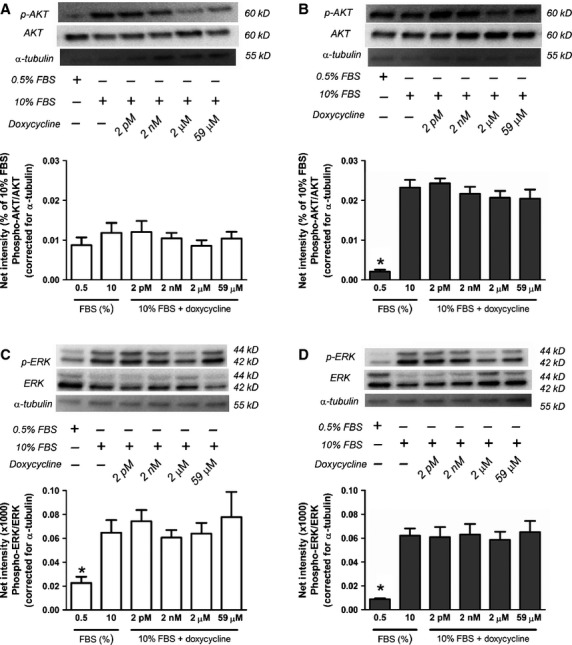
Phospho-AKT levels in (**A**) TSC2-positive MEF (*n* = 4) and (**B**) TSC2-negative MEF (*n* = 4) and phospho-ERK1/2 (p44/42) levels in (**C**) TSC2-positive MEF (*n* = 4) and (**D**) TSC2-negative MEF (*n* = 4) in the presence (+) or absence (−) of doxycycline as indicated. Representative western blots of phospho-AKT, AKT, phospho-ERK1/2 (p44/42), ERK1/2 (p44/42) and α-tubulin are shown above mean data. Data expressed as mean ± SEM, *P ≤ 0.05 repeated measures one-way anova with a Bonferroni post-test (comparison with 10% FBS).

The amount of phospho-FAK was constitutively higher in TSC2-negative MEF compared to TSC2-positive MEF (*n* = 4, ^#^*P* ≤ 0.05, Fig.5). Doxycycline reduced elevated levels of phospho-FAK in TSC2-negative MEF while it had no effect in TSC2-positive MEF (TSC2-negative MEF, *n* = 4, *P ≤ 0.05, Fig.5). Doxycycline also had no effect on the levels of phospho-AKT (*n* = 4, *P* > 0.05, Fig.6A and B) or phospho-ERK1/2 (p44/42) (*n* = 4, *P* > 0.05, Fig.6C and D) in TSC2-positive or TSC2-negative MEF.

### Combined treatment of doxycycline and rapamycin inhibits the rate of wound closure in TSC2-negative MEFs

**Figure 7:**
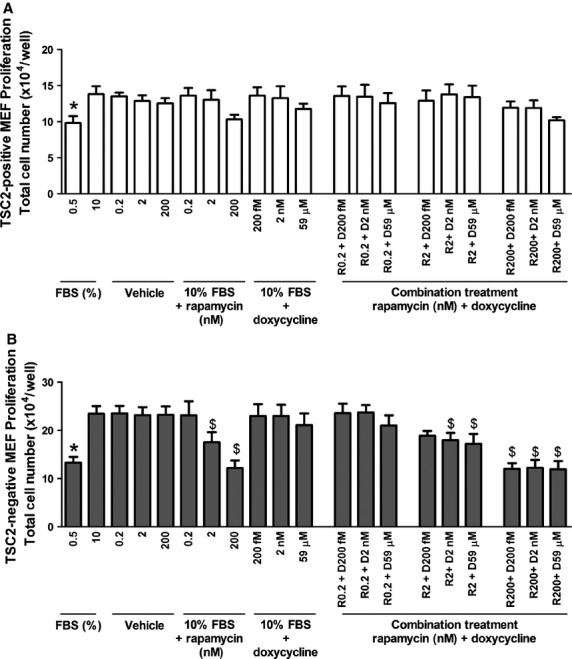
Proliferation of (**A**) TSC2-positive MEF (*n* = 4) and (**B**) TSC2-negative MEF (*n* = 4) treated with doxycycline, vehicle or rapamycin alone, or in combination as indicated. Data expressed as mean ± SEM, *P ≤ 0.05 repeated measures one-way anova compared to FBS ^$^*P* ≤ 0.05 repeated measures one-way anova compared to Vehicle (DMSO) control where appropriate. Bonferroni post-test was used.

**Figure 8:**
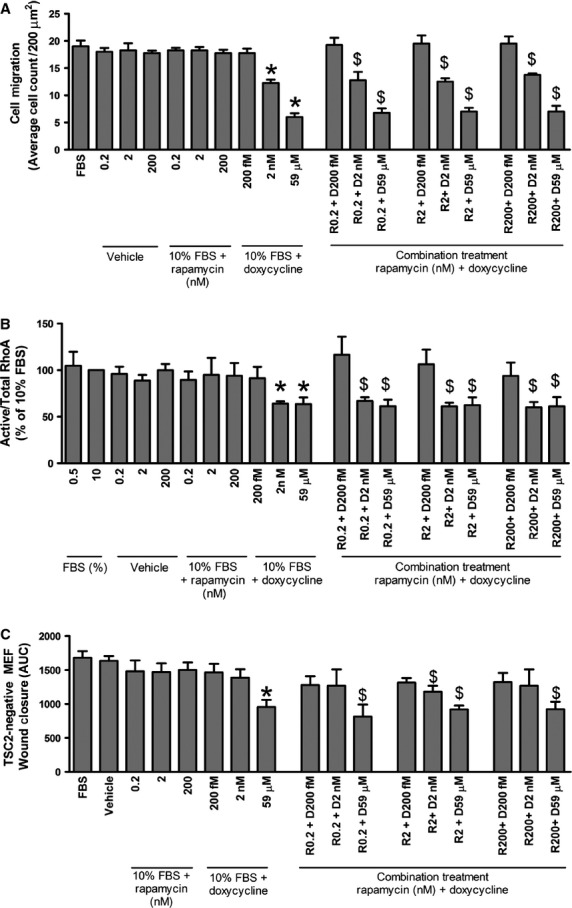
Cell migration of (**A**) TSC2-negative MEF (*n* = 4), treated with doxycycline (D) vehicle or rapamycin (R) alone, or in combination (R+D). Data expressed as mean ± SEM, *P ≤ 0.05 comparison with 10% FBS, ^$^*P* < 0.05 comparison with vehicle control, using repeated measures one-way anova. (**B**) RhoA activity of TSC2-negative MEF (*n* = 6) treated with D, vehicle or R alone or in combination. RhoA activity corrected for total Rho (-A, -B, -C) and expressed as a percentage of 10% FBS ± SEM, *P ≤ 0.05 comparison with 10% FBS, ^$^*P* < 0.05 comparison with vehicle control using repeated measures one-way anova. (**C**) Wound closure of TSC2-negative MEF (*n* = 4) treated with doxycycline, vehicle or rapamycin 2 nM alone or in combination. Data expressed as area under the curve (AUC) over 20 hrs ± SEM, *P ≤ 0.05 comparison with 10% FBS, ^$^*P* < 0.05 comparison with vehicle control, analysed using repeated measures one-way anova as appropriate.

We next assessed the effectiveness of combining rapamycin with doxycycline in inhibiting the proliferation, migration and the ability to close a wound of TSC2-null MEF, as well as its effect on RhoA-GTPase activity. Doxycycline or rapamycin alone or in combination had no effect on the proliferation of TSC2-positive MEF (TSC2-positive, *n* = 4; *P* > 0.05, Fig.7A). Combining doxycycline and rapamycin significantly inhibited TSC2-negative MEF proliferation, migration and RhoA-GTPase activity, however, the degree of inhibition was no greater compared to the individual drugs alone (Cell proliferation, TSC2-negative, *n* = 4; *P* > 0.05, Fig.7B, cell migration, TSC2-negative, *n* = 4; *P* > 0.05, Fig.8A and RhoA activity, *n* = 6, *P* > 0.05, Fig.8B). The combination of doxycycline and rapamycin at sub-maximal concentrations decreased the rate of wound closure in TSC2-negative MEF when compared to the individual effects of doxycycline or rapamycin alone (TSC2-negative, *n* = 4; *P* < 0.05, Fig.8C).

## Discussion

A lack of treatment for LAM has prompted studies to further understand the pathogenic characteristics of the disease, in an attempt to pinpoint possible future therapeutic targets. In this study, we demonstrated the ability of doxycycline to reduce the enhanced migratory capacity of cells deficient for TSC2 through the inhibition of RhoA-GTPase activity and reduced levels of phospho-FAK. In addition, we extended our research to examine the combination of doxycycline and rapamycin and showed this reduced the rate of wound closure and maintained the reduction in migration, proliferation and RhoA-GTPase activity in TSC2-null cells.

As LAM is associated with TSC2 dysfunction, cells deficient for TSC2 such as MEF, 323-MEF and ELT3 cells, have been widely used to enhance our understanding of this disease 47. The use of TSC2-negative MEF and 323-TSC2-null MEF in this study is of particular relevance to LAM as some LAM cells have been reported to be TSC2 negative 7,48,49. In addition, TSC2-negative and 323-TSC2-null MEF have also been shown to exhibit enhanced proliferation and migration, characteristics which are also shared by LAM cells 8,9,21,50.

The dysfunction of TSC2 has been widely demonstrated to be associated with enhanced proliferation of LAM cells 3,11,13,18,45,51. Many studies have focused on controlling this enhanced proliferation through the anti-proliferative properties of rapamycin (an mTORC1 inhibitor) 11,31,52,53. However, it is as important to recognise that the loss of TSC2 function in LAM not only results in the hallmark manifestations of enhanced cell proliferation, but also enhanced cell migration. For this reason, we believe that targeting the increased migratory capacity of cells deficient for TSC2 may be beneficial.

It is well-established that TSC2 plays a prominent role in the regulation of cell migration 9,10,46,51,54. Previous studies that examined cell migration using a transwell migration assay have demonstrated enhanced migratory capacity in cells deficient for TSC2. In this study, we were able to confirm these findings and extend the study to assess the rate at which TSC2-null cells can close a wound. Here, we demonstrated that the rate of wound closure in cells deficient for TSC2 was significantly increased compared to TSC2-positive cells.

In this study, we have demonstrated for the first time that doxycycline can reduce the migratory capabilities and reduce the rate of wound closure of TSC2-negative MEF and 323-TSC2-null MEF. It was of great interest that doxycycline inhibited cell migration at concentrations as low as 20 pM whereas it had no effect on the proliferation of 323-TSC2-positive and 323-TSC2-null MEF. This is in accordance with our previous study that showed doxycycline had no effect on the proliferation of TSC2-positive, TSC2-null MEF or human LAM cells 37. Also in the present study, we showed that proliferation of ELT3 cells was inhibited by doxycycline only at high concentrations and these findings are similar to those of Chang *et al*. who reported that doxycycline at concentrations of 2–20 μM [1–10 μg/ml] had no effect on the proliferation of ELT3 cells but concentrations >49 μM [>25 μg/ml] decreased proliferation, increased apoptosis and altered cell morphology - effects which were caused by doxycycline-induced toxicity 55. Furthermore, we demonstrated rapamycin to be ineffective in the inhibition of migration and confirmed the findings of Goncharova *et al*. who showed rapamycin, at concentrations that significantly abrogated LAM cell proliferation, had no effect on human LAM cell migration 9. Wound closure assays have previously been reported to reflect two crucial processes, cell proliferation and migration 56. However, we have demonstrated in this study that, the ability of TSC2-negative and 323-TSC2-null MEFs to close a wound relies predominantly on migration and not proliferation, as rapamycin had little effect in these assays.

Matrix metalloproteinase -2 and -9 have often been associated with cell migration and invasion; however the concentrations of doxycycline that inhibited cell migration were significantly lower than those we have previously reported for effective MMP-2 inhibition 37. This suggests that, although doxycycline is capable of MMP-2 inhibition, the reduction in migratory capacity demonstrated in this study is not likely to be because of the inhibition of MMPs. It was also interesting to observe that doxycycline was not as effective in the wound closure assay as in the transwell migration assay, where concentrations of only ≥20 μM [10 μg/ml] significantly inhibited wound closure. This may be in part because of other processes involved in the wound closure assay such as cell-cell interactions.

The rapamycin-insensitive mTORC2 pathway regulates cell migration through RhoA-GTPase, and, in this study, we confirmed previous findings that RhoA-GTPase activity in TSC2-negative MEF was higher than in TSC2-positive MEF under basal conditions and in the presence of 10% FBS 9. The mechanism of action through which doxycycline inhibits TSC2-null cell migration was not known, however, this study shows that doxycycline inhibits the migration of TSC2-null cells through the activity of RhoA-GTPase, whereas levels of phospho-p70S6K, phospho-AKT and phospho-ERK1/2 were unaltered. However, the mechanism by which doxycycline inhibits cell migration at the lower concentrations at which RhoA-GTPase activity was not affected (20 pM–200 nM), is likely to be due to targeting other signalling pathways.

Importantly, doxycycline inhibited the activity of RhoA-GTPase in TSC2-negative MEF, while it was unaltered in TSC2-positive MEF. RhoA-GTPase is known to play a prominent role in the regulation and organisation of the cytoskeleton by promoting the assembly of focal adhesions and by activating FAK 58,57, processes which are important for cell motility. We have shown, in this study, that the protein tyrosine kinase FAK is constitutively active in TSC2-negative MEF compared to TSC2-positive MEF, thus supporting the finding of enhanced cell migration in TSC2-negative MEF. Furthermore, doxycycline significantly inhibited levels of phospho-FAK in TSC2-negative MEF while they were unaltered in TSC2-positive MEF. These data show that in TSC2-negative MEF, doxycycline inhibited the activity of RhoA-GTPase at 1 min. This inhibition is then transduced downstream where FAK signalling was inhibited at 4 hrs and subsequently reduced functional migration at 4 hrs. The finding that doxycycline can regulate cell migration and FAK expression supports the findings by Sun *et al*. who showed doxycycline inhibited the adhesion and migration of melanoma cells through the inhibition of FAK expression 59. Although no studies have demonstrated the sustained effects of doxycycline in the reduction in RhoA-GTPase activity, Sun *et al*. reported the effects of doxycycline in wound healing assays and FAK expression in the continued presence of doxycycline up to 12 hrs 58. This suggests that the abrupt changes in RhoA-GTPase activity by doxycycline can influence outcomes of FAK expression and functional migration over a sustained period of time.

It is now accepted that rapamycin alone may not be the most effective way to treat LAM, and investigations into combination therapies are underway 21,54. Here, we explored the effects of combining doxycycline and rapamycin on the migration, proliferation, wound closure and RhoA activity of cells deficient for TSC2. Although combining doxycycline and rapamycin in this study inhibited those cellular parameters, the degree of inhibition was no greater than that of the individual drugs alone. The absence of a greater effect from the combination treatment was not surprising, as each drug works to inhibit independent functional characteristics and signalling pathways. However, it is important to note that the combination of doxycycline and rapamycin was not detrimental to the individual outcome, *i.e*. it did not reduce the degree of inhibition caused by the individual drug treatment. Furthermore, as shown in the wound closure assay, combination treatment with submaximal doxycycline and rapamycin concentrations significantly reduced the rate of wound closure, whereas no effect was observed with individual doxycycline and rapamycin treatments at those concentrations. The mechanisms through which combination therapy reduced wound closure in TSC2-negative MEF were not investigated in this study.

The results from this study suggest that doxycycline may be of potential therapeutic benefit as a treatment for diseases in which TSC2 dysfunction results in enhanced cellular migration. In addition, this study provides preliminary evidence that the combination of doxycycline and rapamycin may lower the dosing requirement for rapamycin, and in turn potentially reduce the side effects associated with chronic rapamycin use.

## Supporting Information

**Figure S1** Basal level (0.5% FBS) and 10% FBS-induced proliferation of TSC2-positive (*n* = 12) and TSC2-negative (*n* = 12) MEF. Data expressed as mean ± SEM, ^#^*P* ≤ 0.05 TSC2-negative versus TSC2-positive cells, repeated measures two-way anova with a Bonferroni post-test.

**Figure S2** Basal level (0.5% FBS) and 10% FBS-induced proliferation of 323-TSC2-positive (*n* = 8) and 323-TSC2-null MEF (*n* = 8). Data expressed as mean ± SEM, ^#^*P* ≤ 0.05 repeated measures two-way anova with a Bonferroni post-test.

**Figure S3** Proliferation of 323-TSC2-positive (*n* = 5) and 323-TSC2-null MEF (*n* = 5), treated with doxycycline. Data expressed as mean ± SEM, ^#^*P* ≤ 0.05 repeated measures one-way anova with a Bonferroni post test.

**Figure S4** Proliferation of ELT3 cells treated with doxycycline. Data expressed as mean ± SEM, **P* ≤ 0.05 compared to 10% FBS using repeated measures one-way anova with a Bonferroni post-test.

**Figure S5** Proliferation of TSC2-positive (white bars, *n* = 5) and TSC2-negative MEF (grey bars, *n* = 5), treated with Y-27632. Data expressed as mean ± SEM, ^#^*P* ≤ 0.05 repeated measures one-way anova with a Bonferroni post test.

**Figure S6** Proliferation of TSC2-positive (*n* = 4) and TSC2-negative MEF (*n* = 4) treated with vehicle (V) or rapamycin. ^#^*P* < 0.05 comparison with TSC2-positive cells. ^$^*P* < 0.05 comparison with vehicle control.

**Figure S7** Proliferation of 323-TSC2-positive MEF (*n* = 4) and 323-TSC2-null MEF (*n* = 4) treated with vehicle (V) or rapamycin. ^#^*P* < 0.05 comparison with TSC2-positive cells. ^$^*P* < 0.05 comparison with vehicle control.

**Figure S8** Proliferation of ELT3 cells treated with vehicle (V) or rapamycin. **P* < 0.05 comparison with 10% FBS. ^$^*P* < 0.05 comparison with vehicle control.
