# Supplementary figures and images for: Doxycycline reduces the migration of tuberous sclerosis complex-2 null cells - effects on RhoA-GTPase and focal adhesion kinase
Source: J Cell Mol Med. 2015 Aug 18;19(11):2633–46. doi: 10.1111/jcmm.12593 (PMC4627568; doi:10.1111/jcmm.12593)

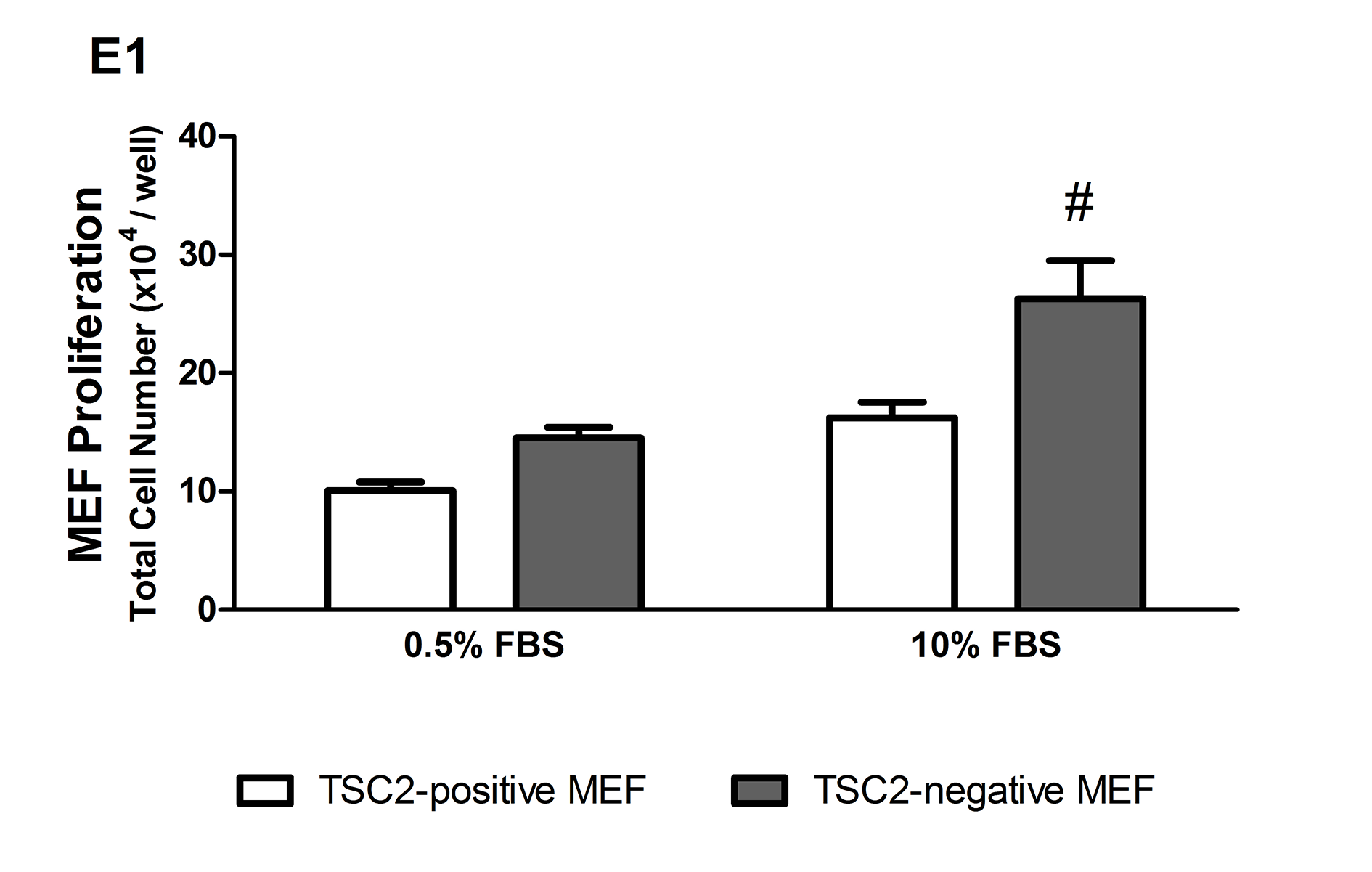

Supplement: Supplementary file 1 — Figure S1 Basal level (0.5% FBS) and 10% FBS-induced proliferation of TSC2-positive (n = 12) and TSC2-negative (n = 12) MEF. Data expressed as mean ± SEM, #P ≤ 0.05 TSC2-negative versus TSC2-positive cells, repeated measures two-way anova with a Bonferroni post-test. [file jcmm0019-2633-sd1.tif]

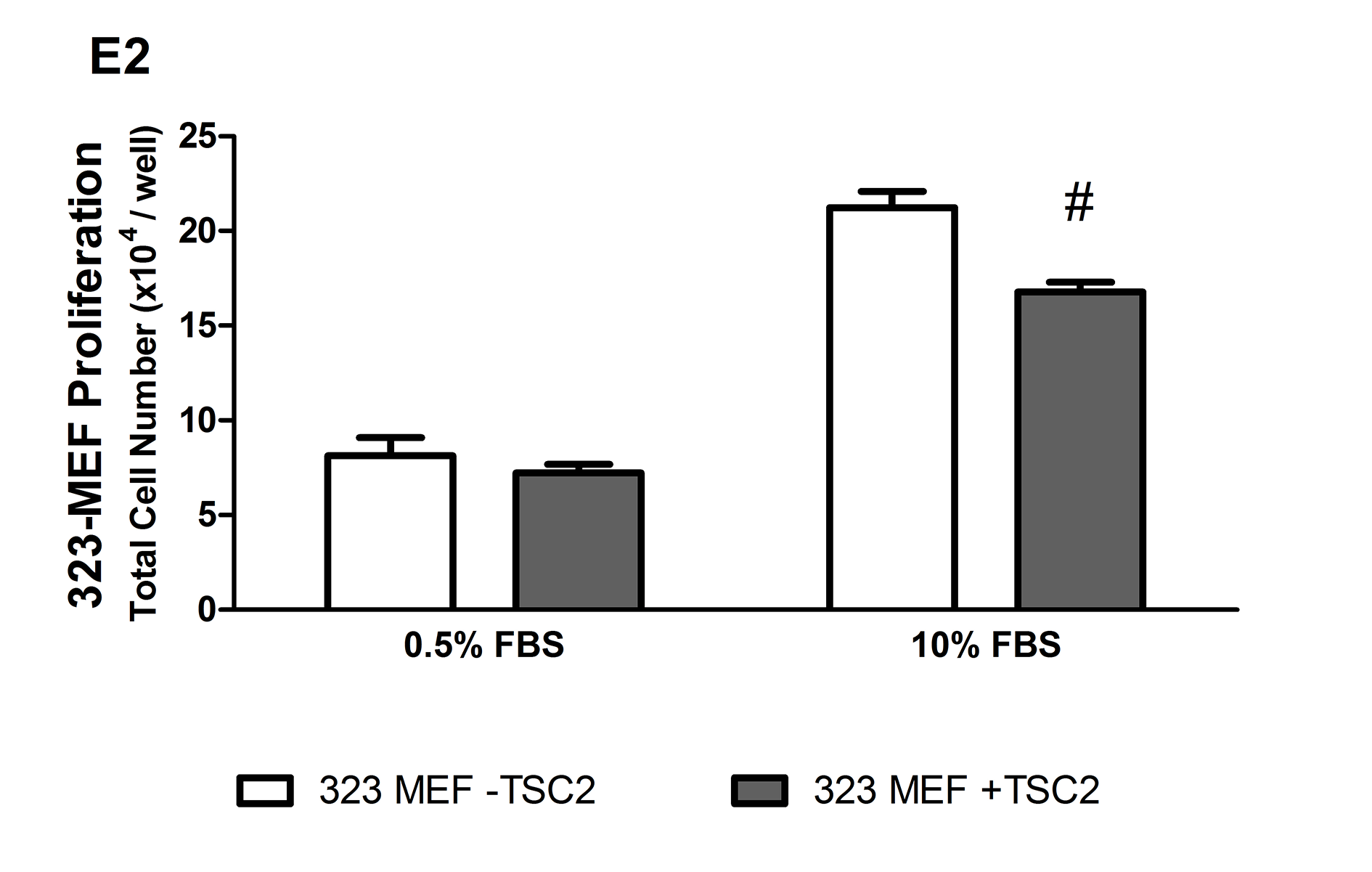

Supplement: Supplementary file 2 — Figure S2 Basal level (0.5% FBS) and 10% FBS-induced proliferation of 323-TSC2-positive (n = 8) and 323-TSC2-null MEF (n = 8). Data expressed as mean ± SEM, #P ≤ 0.05 repeated measures two-way anova with a Bonferroni post-test. [file jcmm0019-2633-sd2.tif]

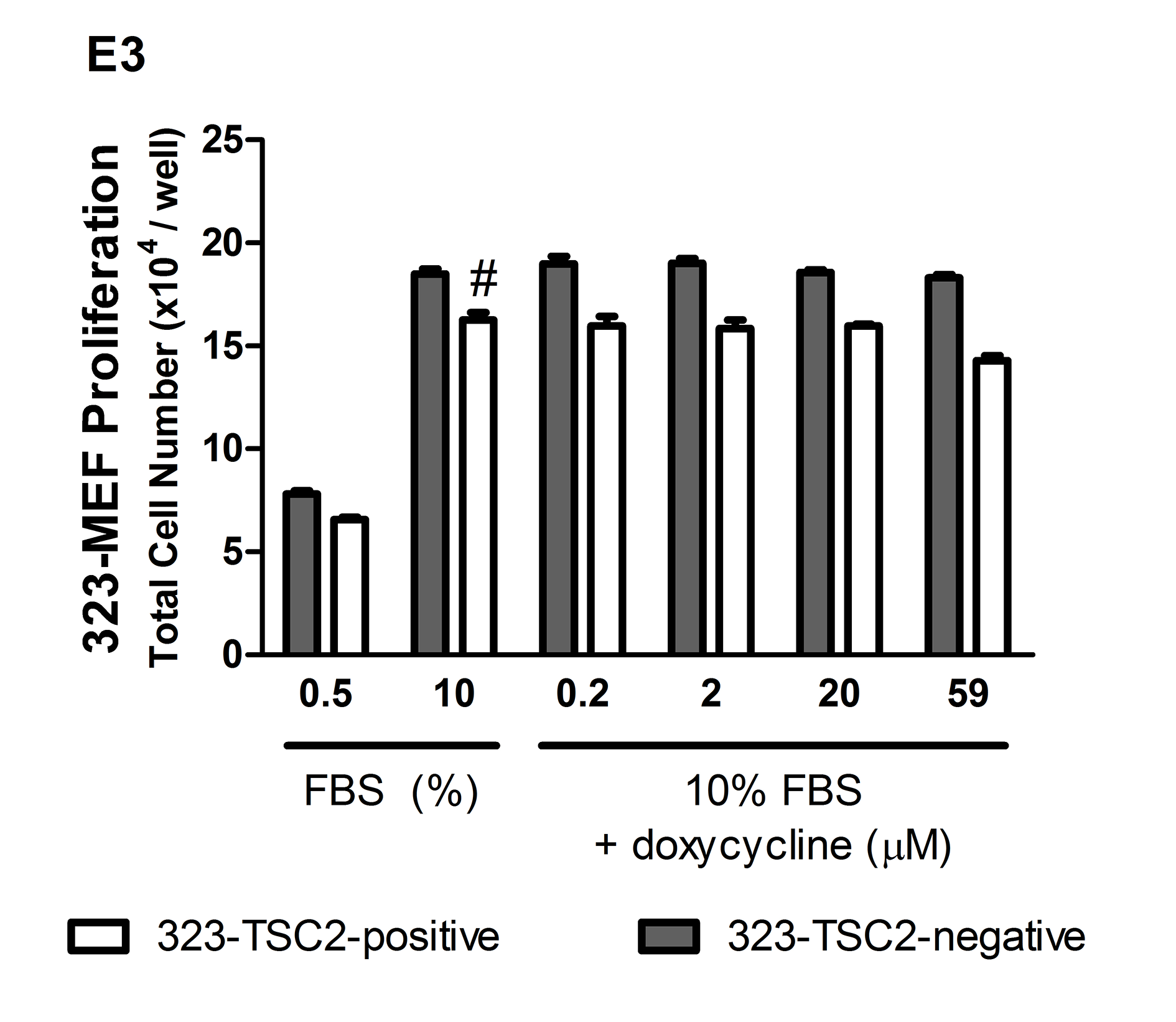

Supplement: Supplementary file 3 — Figure S3 Proliferation of 323-TSC2-positive (n = 5) and 323-TSC2-null MEF (n = 5), treated with doxycycline. Data expressed as mean ± SEM, #P ≤ 0.05 repeated measures one-way anova with a Bonferroni post test. [file jcmm0019-2633-sd3.tif]

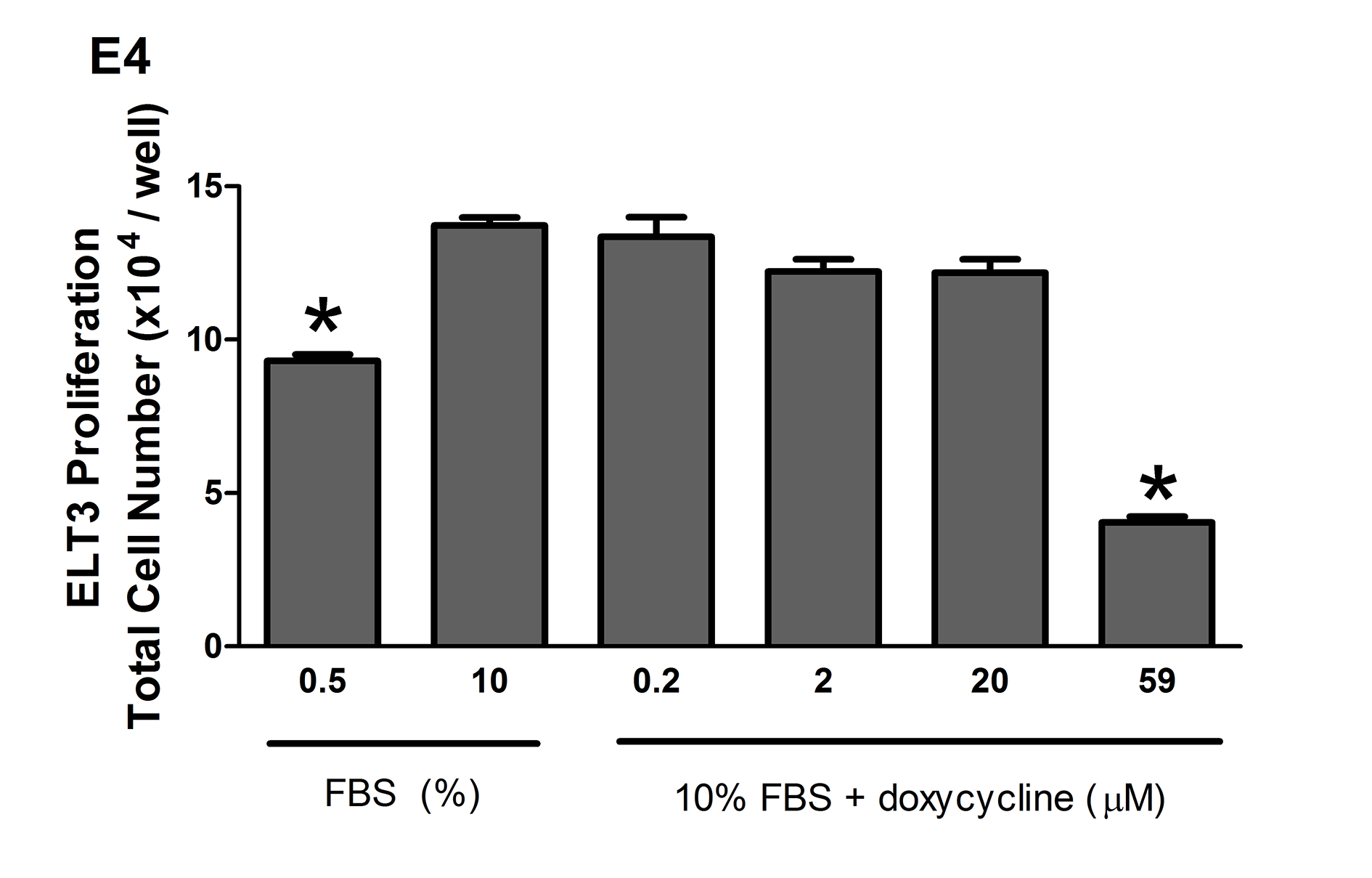

Supplement: Supplementary file 4 — Figure S4 Proliferation of ELT3 cells treated with doxycycline. Data expressed as mean ± SEM, *P ≤ 0.05 compared to 10% FBS using repeated measures one-way anova with a Bonferroni post-test. [file jcmm0019-2633-sd4.tif]

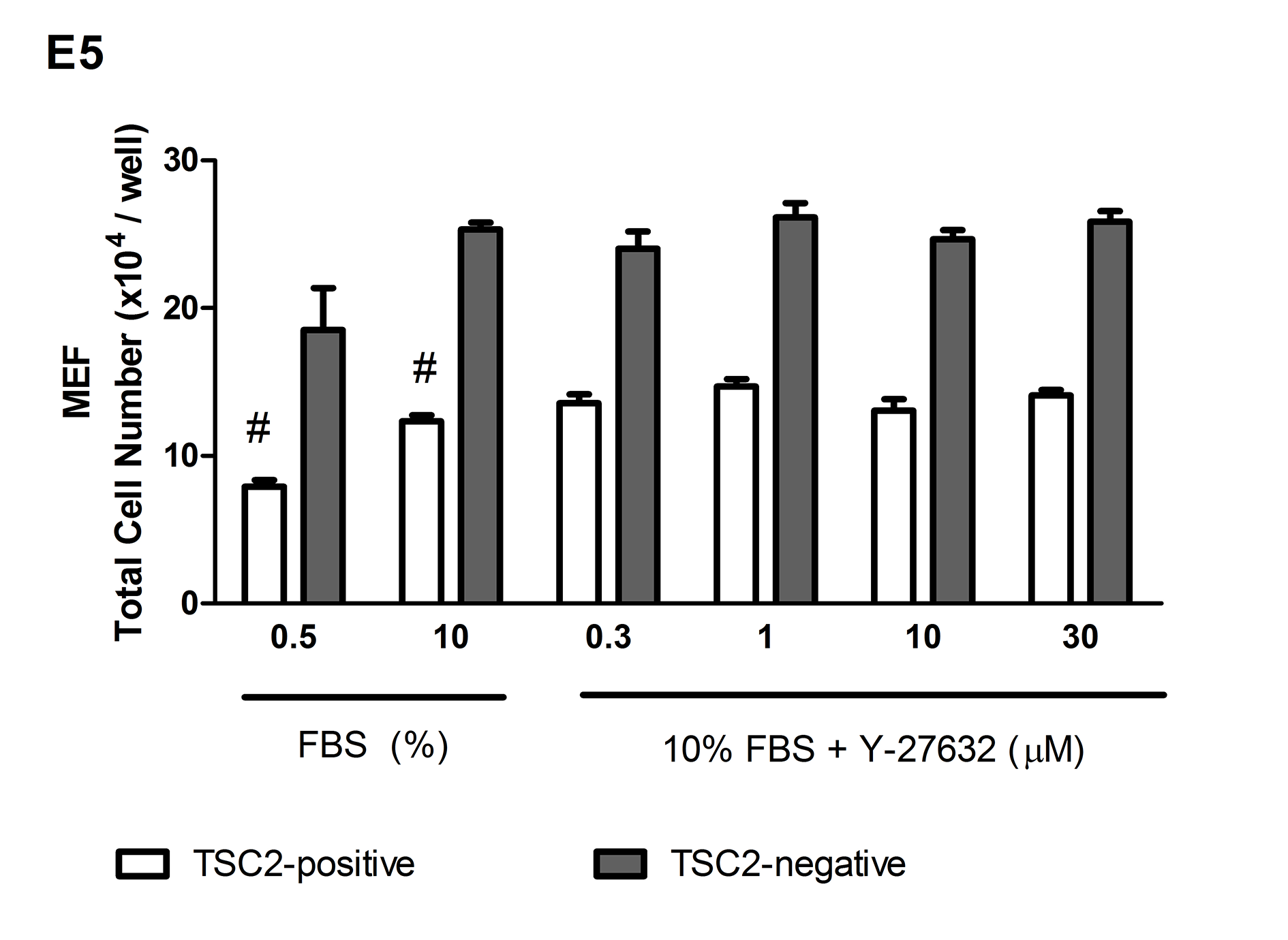

Supplement: Supplementary file 5 — Figure S5 Proliferation of TSC2-positive (white bars, n = 5) and TSC2-negative MEF (grey bars, n = 5), treated with Y-27632. Data expressed as mean ± SEM, #P ≤ 0.05 repeated measures one-way anova with a Bonferroni post test. [file jcmm0019-2633-sd5.tif]

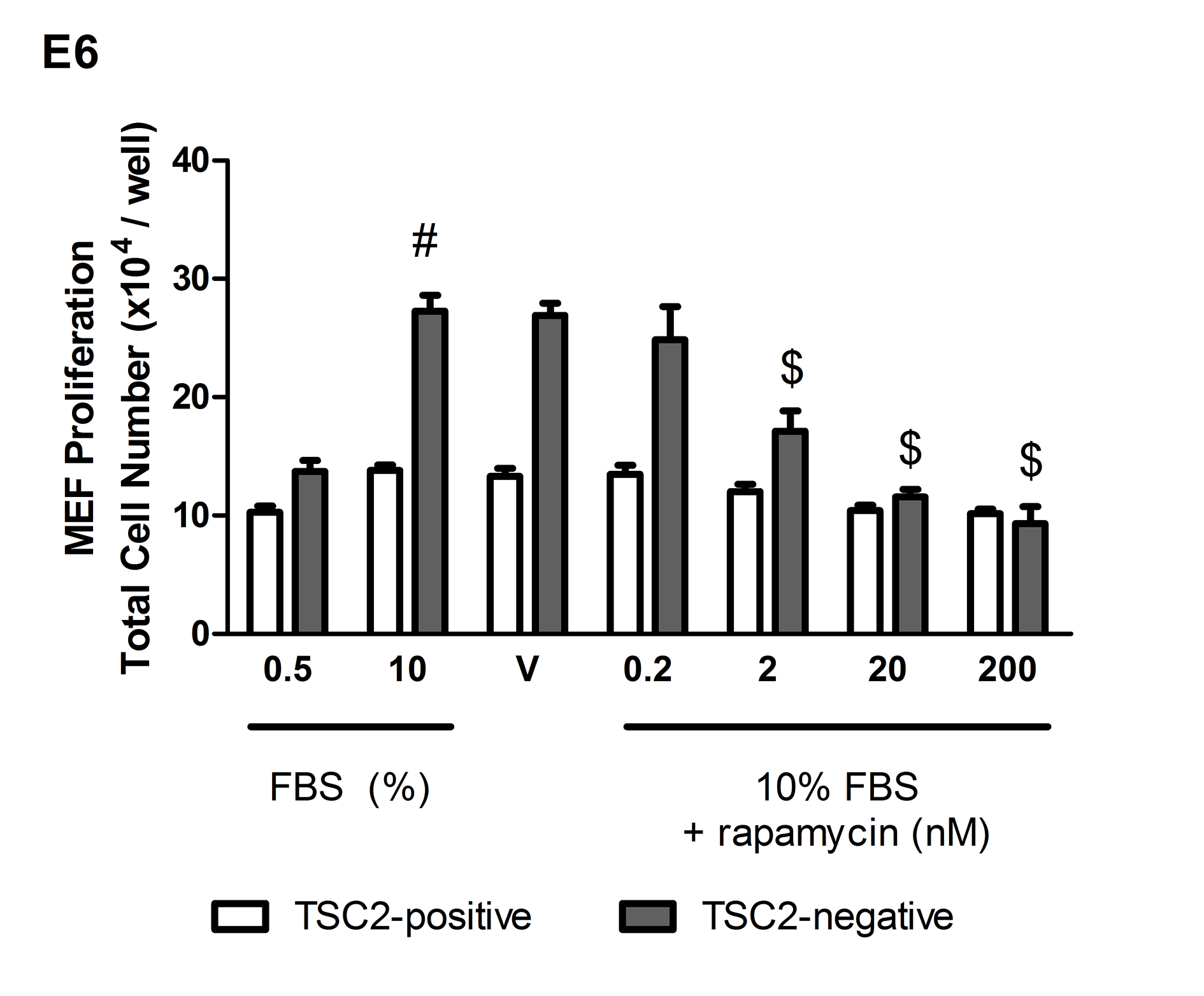

Supplement: Supplementary file 6 — Figure S6 Proliferation of TSC2-positive (n = 4) and TSC2-negative MEF (n = 4) treated with vehicle (V) or rapamycin. #P < 0.05 comparison with TSC2-positive cells. $P < 0.05 comparison with vehicle control. [file jcmm0019-2633-sd6.tif]

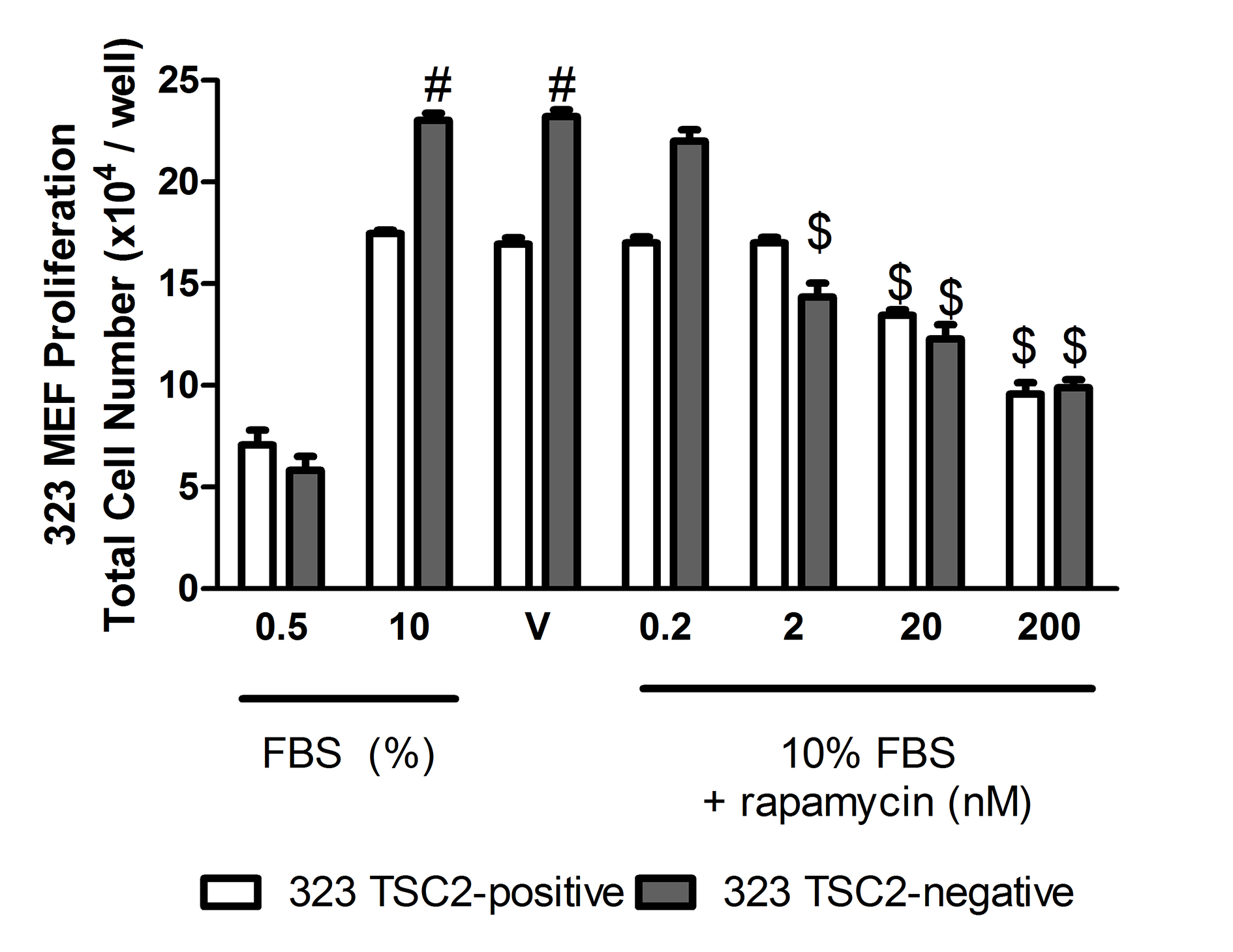

Supplement: Supplementary file 7 — Figure S7 Proliferation of 323-TSC2-positive MEF (n = 4) and 323-TSC2-null MEF (n = 4) treated with vehicle (V) or rapamycin. #P < 0.05 comparison with TSC2-positive cells. $P < 0.05 comparison with vehicle control. [file jcmm0019-2633-sd7.tif]

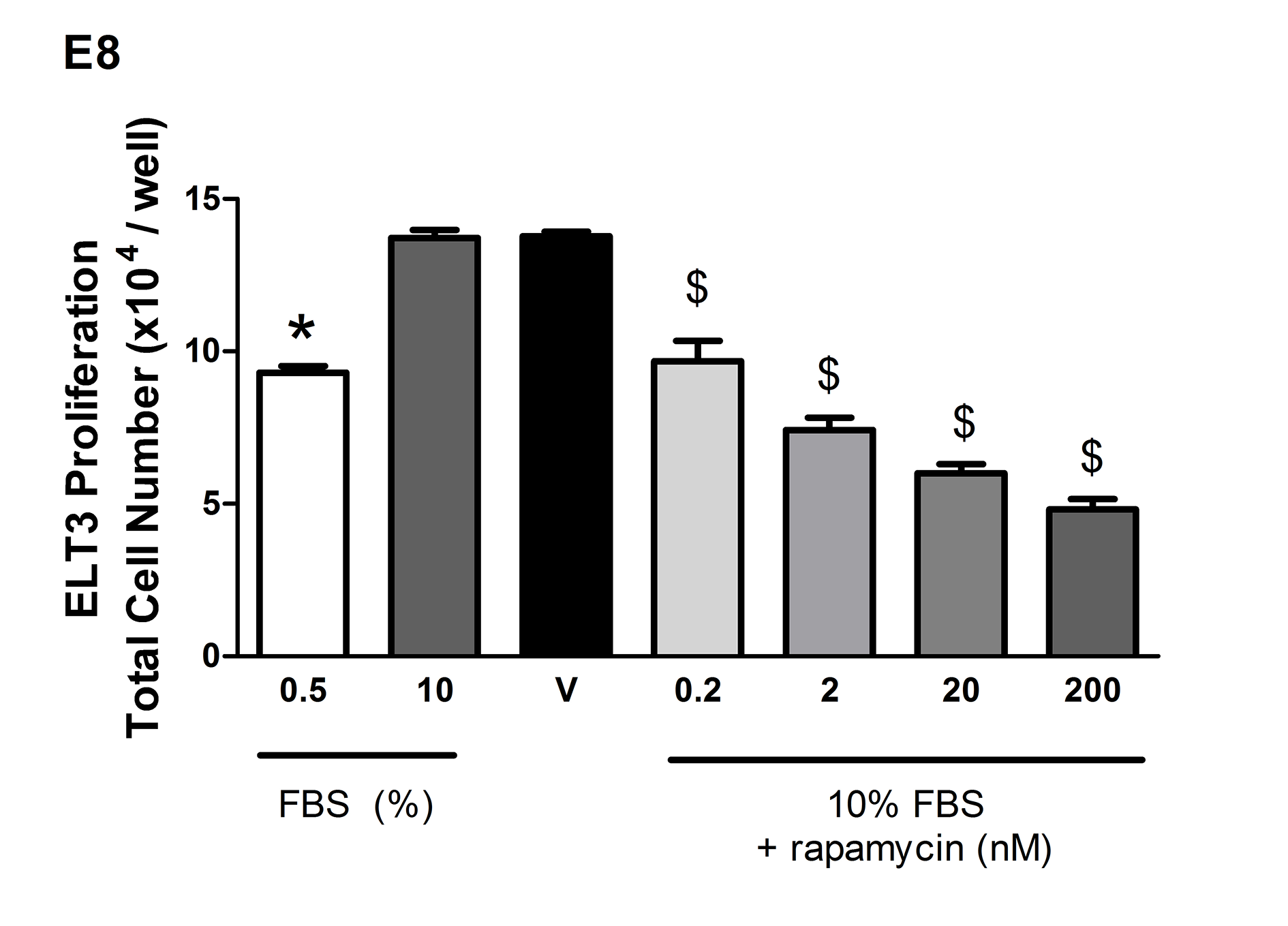

Supplement: Supplementary file 8 — Figure S8 Proliferation of ELT3 cells treated with vehicle (V) or rapamycin. *P < 0.05 comparison with 10% FBS. $P < 0.05 comparison with vehicle control. [file jcmm0019-2633-sd8.tif]
